# Rethinking origin licensing

**DOI:** 10.7554/eLife.24052

**Published:** 2017-01-19

**Authors:** Stephen P Bell

**Affiliations:** Department of Biology, Howard Hughes Medical Institute, Massachusetts Institute of Technology, Cambridge, United Statesspbell@mit.edu

**Keywords:** DNA replication, CDC6, ORC, MCM2-7, CDT1, Human

## Abstract

Human cells that lack a subunit in their origin recognition complex are viable, which suggests the existence of alternative mechanisms to initiate DNA replication.

**Related research article** Shibata E, Kiran M, Shibata Y, Singh S, Kiran S, Dutta A. 2016. Two subunits of human ORC are dispensable for DNA replication and proliferation. *eLife* **5**:e19084. doi: 10.7554/eLife.19084

When a eukaryotic cell divides it must replicate its DNA, and the process of replication starts at hundreds or thousands of separate sites called origins of replication. The first step in replication, which is known as origin licensing, involves the recruitment of a ring-shaped helicase enzyme called MCM2-7 such that it encircles each origin of replication. The fact that this enzyme and three other proteins required for origin licensing – the origin recognition complex, CDC6 and CDT1 – are conserved throughout eukaryotic evolution confirms the central importance of origin licensing (Makarova and Koonin, 2013).

It is thought that the origin recognition complex, which consists of six proteins called ORC1-ORC6, performs two roles during origin licensing (O'Donnell et al., 2013). First, by binding to DNA and histone proteins, it selects the sites where origin licensing takes place. Second, in conjunction with CDC6, it recruits the MCM2-7 helicase (which is bound to a CDT1 protein) to each origin of replication. Structural studies indicate that CDC6 binds to the origin recognition complex to form a six-subunit protein ring (made up of CDC6 and ORC1-ORC5) around the DNA at the origin of replication (Figure 1A; Bleichert et al., 2015; Sun et al., 2013). This ring then binds to the MCM2-7 helicase such that the latter encircles the DNA adjacent to the origin of replication (Sun et al., 2013). Subsequent events activate MCM2-7, allowing it to separate the DNA strands so that they can be copied (Bell and Labib, 2016).

**Figure 1.**
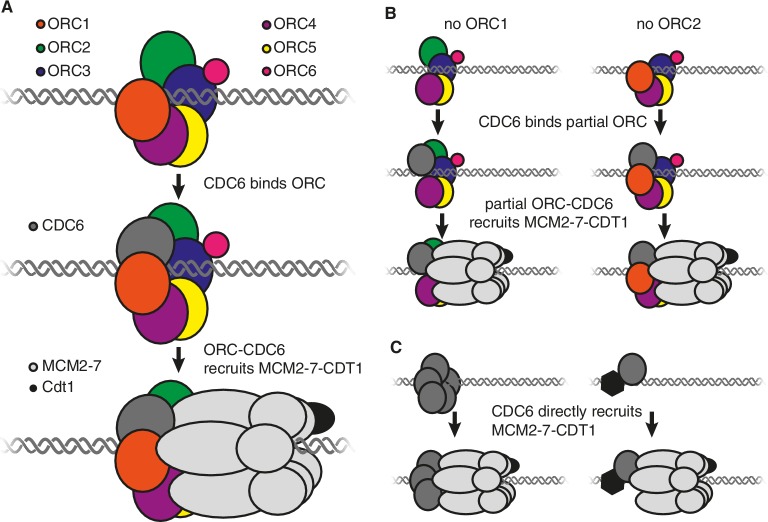
Models showing the roles of the origin recognition complex (ORC) during origin licensing. (**A**) In eukaryotic cells under normal conditions origin licensing starts with the ORC1-ORC5 subunits of ORC partially encircling the origin of replication. CDC6 (dark gray) then binds to ORC1 (red) and ORC2 (green) to complete a protein ring around the DNA. The ORC-CDC6 complex interacts with a ring-shaped MCM2-7 helicase (light gray) such that the helicase (which is bound to the CDT1 protein; black) encircles the adjacent DNA. (**B**) A model for origin licensing by an origin recognition complex that lacks ORC1 or ORC2. This partial ORC still binds to DNA, and CDC6 binds to either ORC1 or ORC2 to form a five-subunit partial ring around the DNA. This new complex retains the ability to recruit MCM2-7 and CDT1. (**C**) A model for how origin licensing could occur in the absence of the origin recognition complex. CDC6 either directly (left) or indirectly (by binding a different DNA-bound protein; right) binds to DNA and recruits MCM2-7 and CDT1 to the origin of replication.

Given the importance of origin licensing it is not surprising that deleting any of the ORC genes is lethal to yeast cells and fruit flies, and depletion of ORC prevents DNA replication in frog egg extracts (Li and Stillman, 2012). Now, in eLife, Anindya Dutta and colleagues at the University of Virginia School of Medicine – including Etsuko Shibata as first author – report that human cells with loss-of-function mutations in the genes for ORC1 or ORC2 are viable (Shibata et al., 2016). This is a surprising observation given the importance of these genes in other organisms.

Although the cells with a mutant form of the ORC1 or ORC2 gene have phenotypes that are consistent with replication defects, the impact on cell division is limited. Regardless of which gene was mutated, the resulting cells maintained a proliferation rate of half that seen in normal cells and no difference was seen in the time they spent replicating the DNA. Mapping the origins of replication showed that the mutant cells had approximately half as many of these sites as normal cells. Consistent with the previously defined roles of the origin recognition complex, the amount of DNA-associated MCM2-7 was reduced in both mutant cell lines, and the origins of replication only partially overlapped.

How can cells live when they lack a subunit of the origin recognition complex? One possibility is that the mutant alleles do not completely eliminate the subunit targeted by the mutation. Although no residual ORC1 or ORC2 proteins were detected, Shibata et al. estimate that up to 1% of the proteins could still have been present in the cells. If residual ORC1 or ORC2 explains the viability of the mutant cells, each remaining origin recognition complex would have to load more than 35 helicases over 3.5 million DNA base pairs. Furthermore, although a lower abundance is consistent with the reduced number of origins of replication observed in the mutant cells, it does not explain why 60% of these sites are at new locations.

Alternatively, a partial origin recognition complex could be sufficient for origin licensing. In this regard, it is noteworthy that ORC1 and ORC2 are at either end of the five ORC subunits that form a ring around the DNA and each has a binding site for CDC6 (Figure 1A; Sun et al., 2013). The loss of either of these subunits would leave a complex containing just four subunits but, crucially, each of these partial complexes would retain the ability to bind to CDC6. Therefore, it is possible that an origin recognition complex that lacks ORC1 or ORC2 could still form a ring (from four ORC subunits and CDC6) that retains the ability to bind strongly to DNA, CDC6 and MCM2-7 (Figure 1B).

Several observations are consistent with the possibility that such a partial complex can perform origin licensing. Mutations that reduced the production of ORC5 or CDC6 decreased DNA synthesis in ORC1 or ORC2 mutants, which indicates that both ORC5 and CDC6 are still involved in DNA replication in the absence of ORC1 or ORC2. In contrast to ORC1 and ORC2, Shibata et al. were unable to obtain viable mutations that prevented the production of the ORC4 or ORC5 proteins. These mutations would break the ring into multiple segments and the inability to obtain these mutants suggests that smaller ORC sub-complexes are non-functional. Finally, a partial origin recognition complex would be expected to have similar but not identical DNA binding properties to the full complex, and so is consistent with the partial overlap in the locations of the origins of replication seen between mutant and non-mutant cells.

It is also possible that the entire origin recognition complex is dispensable. If so, then another origin licensing mechanism must occur that still involves CDC6. For example, one or more CDC6 proteins could directly or indirectly bind to DNA to recruit MCM2-7 (Figure 1C). Although there is no evidence that CDC6 intrinsically binds to DNA, there is evidence that tethering CDC6 to DNA is sufficient to cause the adjacent DNA to be replicated (Takeda et al., 2005), raising the possibility that other proteins could recruit CDC6. In addition, mutant cells that lacked ORC1 adapted to grow more rapidly over time, possibly reflecting the emergence of mutations that promote an alternative mechanism of origin licensing.

Future studies will be required to distinguish between these possibilities. For example, it will be important to determine if it is possible to simultaneously mutate ORC1 and ORC2 in viable cells. If so, this finding would support an ORC-independent model for CDC6 recruitment and origin licensing as the remaining ORC subunits would lack CDC6 binding sites. If not, then a model in which a partial origin recognition complex can perform origin licensing would be more likely. Regardless of the eventual mechanism, these studies reveal a resilience of the human DNA replication machinery to mutation that could facilitate growth as cells mutate during cancerous transformation.
